# Novel 3D printed partial talar surface prosthesis for osteochondral lesions of the talar dome: A 2-year case report

**DOI:** 10.1097/MD.0000000000044226

**Published:** 2025-08-29

**Authors:** Zhi Zou, Chuntao Xu, Zehui Jiang, Lin Zou

**Affiliations:** a Department of Orthopedics, The 960th Hospital of PLA, Jinan, China.

**Keywords:** 3D printed prosthesis, osteochondral lesions of the talar dome, partial talar surface replacement

## Abstract

**Rationale:**

Surgical treatment of osteochondral lesions of the talar dome (OLTD), for lesions larger than 15 mm^2^, surgical treatment remains challenging. To solve these problems, we presented a 3D-printed partial talar surface replacement (PTSR) technique for treating OLTD with severe lesions.

**Patient concerns:**

A 23-year-old male patient with OLTD experienced persistent pain for more than a year (Berndt and Harty classification V). Due to the pain, the patient was not allowed to walk long distances or engage in any physical activities.

**Diagnoses:**

Preoperative clinical examination and imaging (computed tomography and magnetic resonance imaging) confirmed the diagnosis of OLTD and Berndt and Harty classification V.

**Interventions:**

The patient underwent a 3D-printed PTSR surgical procedure.

**Outcomes:**

Follow-up at 2 years after surgery showed good ankle function, with no pain or complications. The Visual Analog Scale score decreased from 5 to 1. The American Orthopedic Foot & Ankle Society scores improved from 65 to 90.

**Lessons:**

3D-printed PTSR is a new surgical method for OLTD with a larger damage area that can provide satisfactory outcomes. Definitive and convincing conclusions are difficult to draw owing to the limited number of cases in this study. Another limitation is the follow-up period, which was 2 years. In addition, medium- and long-term follow-up is required. For this patient, we planned an annual follow-up, which included physical evaluation and radiography. In the future, a prospective randomized controlled study should be conducted to manage the clinical surgical treatment of OLTDs, including metal partial replacements.

## 1. Introduction

Osteochondral lesions of talus (OLT) is a frequently observed condition in ankle injuries.^[1]^ OLT can be attributed to both traumatic and non-traumatic factors. Trauma is considered to be the main cause of osteochondral lesions. It has been observed that approximately 50% of ankle sprains and over 70% of ankle fractures result in OLT.^[2]^

Several studies have shown that most OLTD are located in the talar dome. OLTD are primarily related to trauma.^[3]^ Flick and Gould^[4]^ reported a history of trauma in 98% of lateral and 70% of medial lesions.

The causes of OLTs are diverse. Non-traumatic OLT can be caused by various factors, such as blood vessel or synovial injury, ligament relaxation, osteotic disorder, minimally invasive injury, embolism disease, chronic ankle instability, genetic factors, spontaneous necrosis, physical disorders, hormonal disorders, congenital factors, application of hormones, and alcohol abuse.^[5,6]^

Patients with OLT may have subtalar cysts or even subchondral osteonecrosis.^[7]^ Therefore, the treatments of OLTD combined with subchondral cysts of the talus are challenges in foot and ankle surgery. Advances have been made in the treatment of OLTD.^[8–10]^ Cartilage repair strategies such as arthroscopic bone marrow stimulation and microfracture. Cartilage regeneration strategies such as autologous chondrocyte implantation, matrix-induced autologous chondrocyte implantation, autologous collagen-induced chondrogenesis, and platelet-rich plasma. Additionally, cartilage replacement strategies such as articular cartilage allograft have been used to treat the sever osteochondral lesions.^[11]^ Due to the allograft cartilage sources are relative and the application is initial stage of development, cartilage replacement strategies such as articular cartilage allograft are seldom used in China.^[12]^ With the advancement of 3D printing prosthesis technology, many researches shown to be successful in treating the bone defects and getting good function recovery.^[13,14]^ We introduced a new 3D printed partial talar surface replacement (PTSR) technique in treating an OLTDs > 15 mm^2^. The osteochondral lesions area was replaced with 3D printed prosthesis to relieve pain. To our knowledge, this is the first published report of the treatment of OLTD and the 3D printed partial talar surface prosthesis was approved by the National Patent Office of China (Patent Number: ZL 2023 2 08488815.X).

In this study, we reported the preliminary results of partial talar replacement for the treatment of OLTD.

## 2. Case report

**Figure 1.**
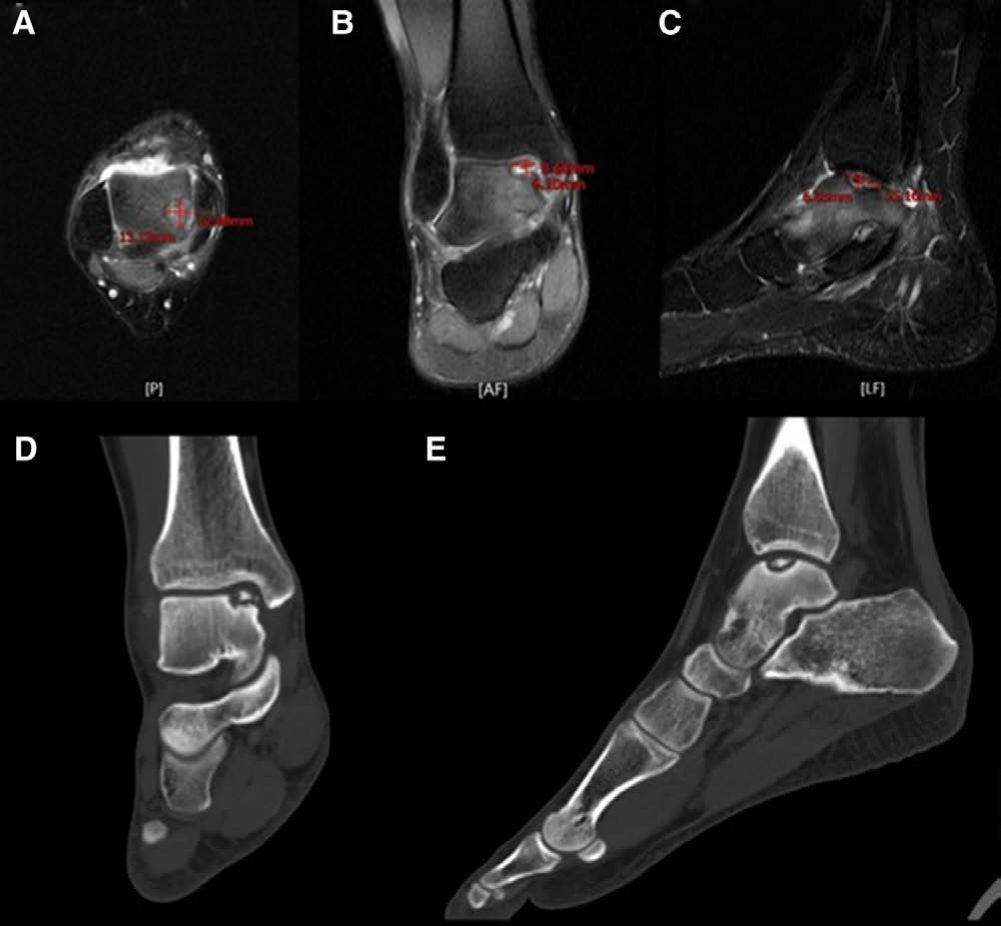
MRI, indicating a medial OCLT involving the dome of the talus (A–C); computed tomography of right ankle demo demonstrating the size and area of talus (D, E).

A 23-year-old male presented to the clinic complaining of chronic right ankle pain 1 year after an ankle sprain. The patient reported self-treatment with rest for a week. He continued to exercise; however, he reported that his ankle remained unstable and had persistent pain since then. When the pain had gradually increased over the past 4 months he was referred to the outpatient department, in which a magnetic resonance scan revealed OLT (Fig. 1). Physical examination revealed tenderness on palpation along the anteromedial ankle. Ankle CT revealed a cystic lesion of the medial talar dome. MRI shows an approximately 15 mm × 12 mm × 6.7 mm area with osteochondral lesions of the medial talar dome, Berndt and Harty classification V (Fig. 1).

After conservative treatment which walking just for daily life and nonsteroidal anti-inflammatory drug more than 3 months, the patient still experienced persistent pain and opted for surgery.

**Figure 2.**
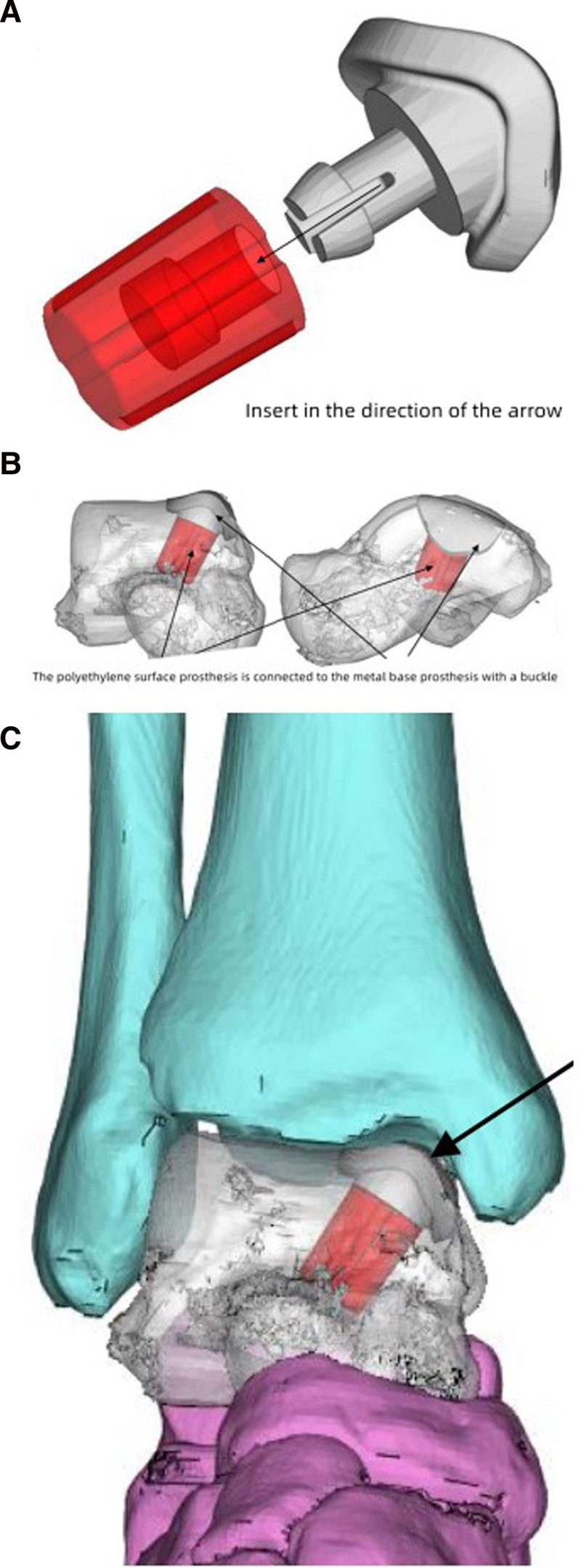
The design of 3D printed prosthesis (A). The installation mode and position of 3D printed prosthesis in talus (B). The view of ankle after installation of prosthesis (C).

Preoperative preparation: the personalized design of the talar surface prosthesis was designed by our team according to the size of the patient’s own osteochondral lesions area. Our team Commissioned Shanghai THYTEC, whose products met the Chinese Food and Drug Administration standards to produce 3D printed prosthesis. The size of the talar surface prosthesis was consistent with the size of the OLT area. Data acquisition and prosthesis printing procedures were conducted as follows: CT (256-layer) data of the patient’s affected and healthy sides were collected, with a thickness of 1 mm. Mimics software was used for 3D modeling, with healthy CT data serving as a reference image. After exporting the data, the Wrap Reverse software was employed to repair the surface and adjust the appearance and size of the prosthesis. The Creo 3D software was used to design the nail hole position and alignment plane for subsequent machining. The articular surface was made of ultra-high molecular weight polyethylene (UHMWPE), whereas the metal base was composed of a titanium alloy porous structure. The polyethylene surface prosthesis was connected to the base using a buckle (Fig. 2). The Arcam printing equipment utilizes a porous structure for the overall printing of the bone contact surface with a porosity range of 60% to 70%.

**Figure 3.**
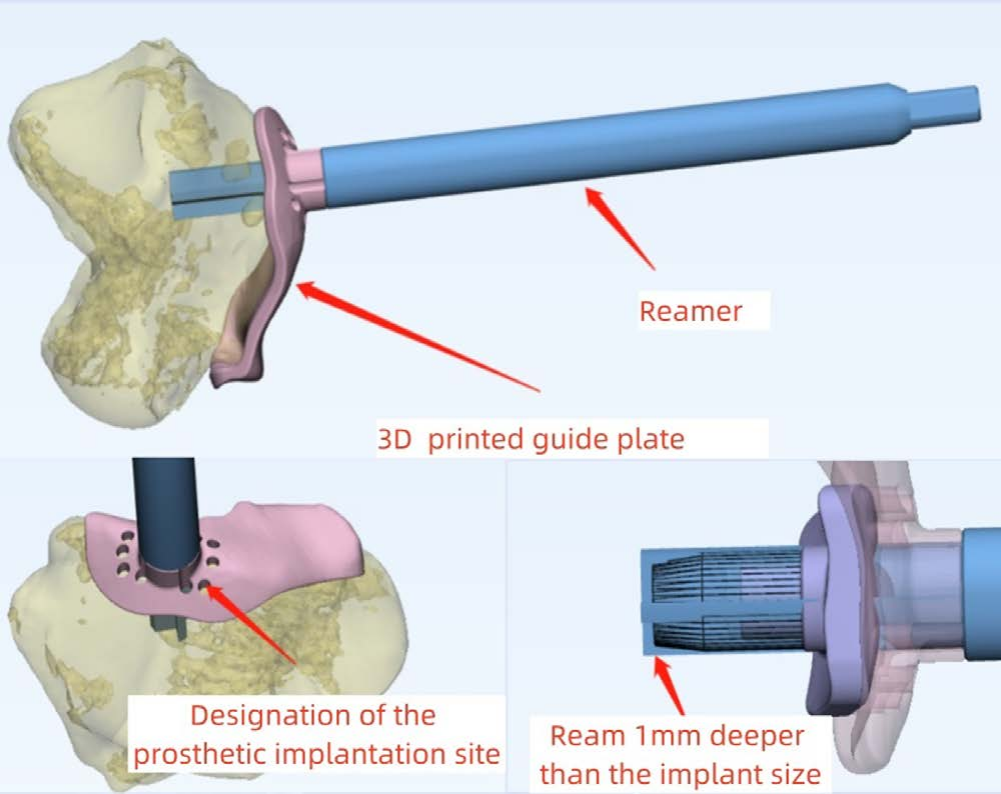
Mark area and removed the marked area unnecessary bone.

**Figure 4.**
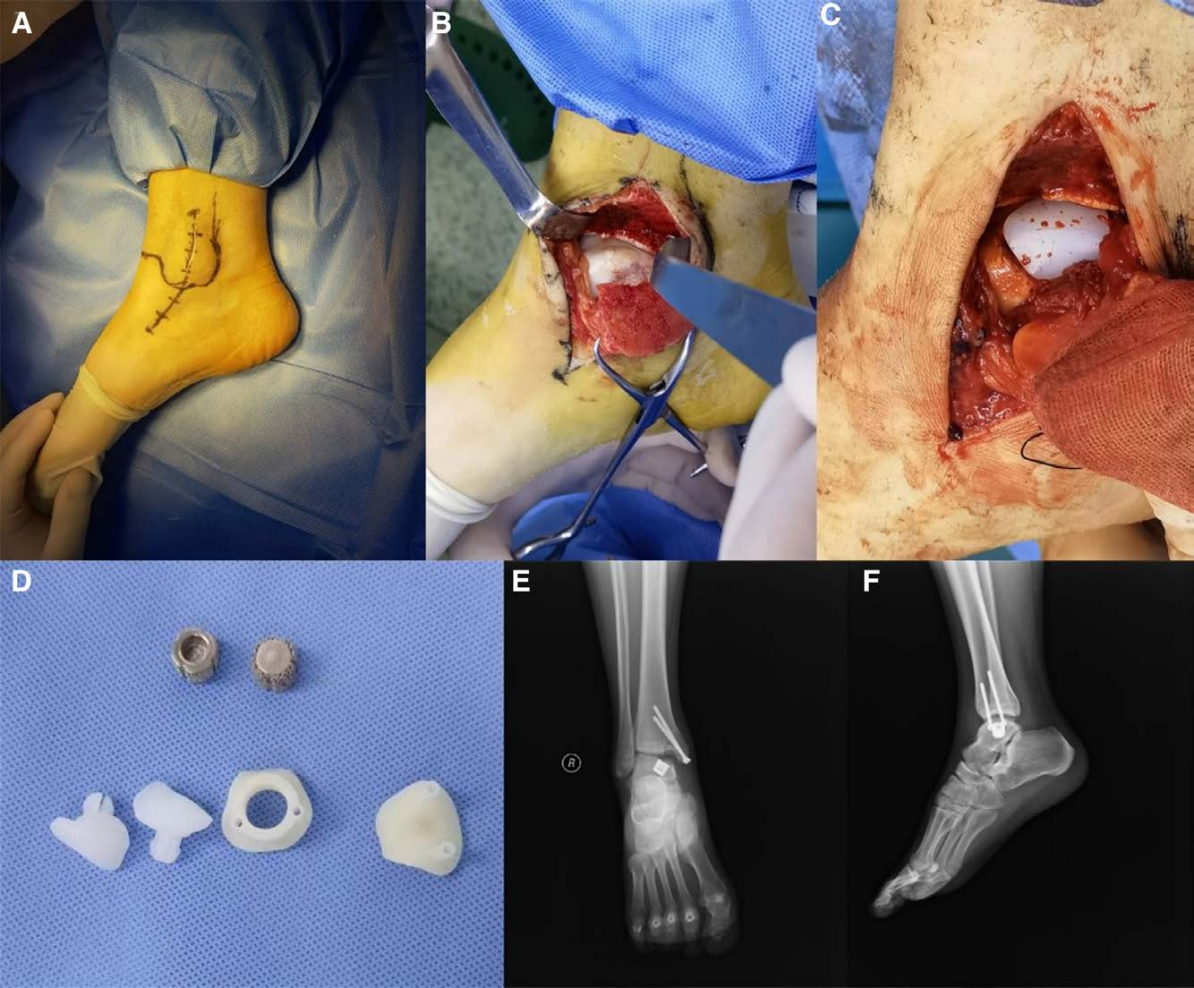
The lesion area of the talus was then clearly exposed via a medial malleolus osteotomy approach. (A) The lesion area of the talus. (B) The view after installation of prosthesis. (C) The 3D-printed prosthesis consists of polyethylene surface prosthesis and titanium alloy porous bone trabecular prosthesis (D). X-rays after Partial talar surface replacement (E, F).

Surgical techniques: the patient was placed in the supine position on the operating table. After the induction of general anesthesia, the lesion area of the talus was clearly exposed through the medial malleolus osteotomy approach. First, a 3D printed guide plate was placed in the lesion area and the prosthesis position could also be marked by the pore through the channel (Fig. 3). Removed the guide plate, removed the marked area unnecessary bone until the prosthesis well matched with surrounding cartilage. Implanted the base prosthesis and polythene surface prosthesis, showed that the talar surface prosthesis matched well. Finally, medial malleolar osteotomy bone pieces were fixed. Radiography was used to determine the reduction of the medial malleolus. The incision was closed. Passive ankle range of motion was measured near-normal. The operation lasted approximately 2 hours (Fig. 4).

**Figure 5.**
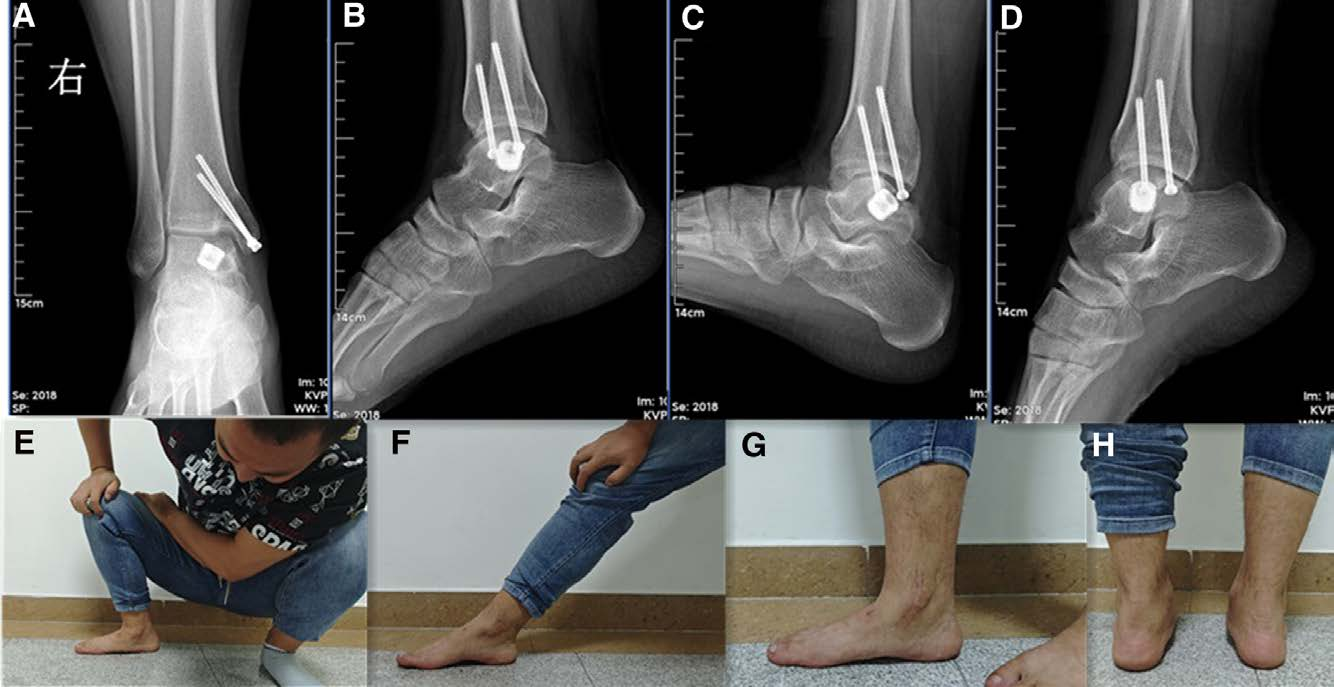
X-rays after partial talar surface replacement after 2 years of surgery (A–D). The function of ankle after 2 years of surgery (E–H).

Postoperative management: the patient remained non-weight-bearing for 6 weeks, allowed flexion and extension. After 6 weeks, the patient was remained partially weight-bearing. Three months postoperatively, the patient began physical therapy and continued not to participate in high-impact activity. At 4 months, the patient began to progress with the activity as tolerated. At 24 months, the patient underwent preoperative activities without discomfort (Fig. 5).

This study was approved by the Institutional Ethics Committee of [The 960th Hospital of PLA] (Approval No. 093), and provided signed informed consent.

## 3. Results

The 3D printed PTSR is a new surgical method for OLTD with a larger damage area provides satisfactory outcomes. No postoperative complications were noted. The patient got reviewed at 1, 3, 6, 12, and 24 months of follow-up. With regard to range of motion, the patient showed an improvement in active dorsiflexion. The Visual Analog Scale score significantly improved postoperatively, from 5 to 1. The American Orthopedic Foot & Ankle Society scores improved from 65 to 90. The patient returned to a normal life. The patient told us doing some exercise activity like jogging and even climbing is allowed and he had no discomfort from the last visit. These results indicate that PTSR in the affected ankle was successful.

## 4. Discussion

### 4.1. Surgical procedures and outcomes

Multiple surgical options are available, and their choice depends on the stage, location, and size of the lesion. Smaller lesions are usually treated using arthroscopic methods. However, chondrocyte transplantation or cartilage grafting transplantation may be considered if the lesion > 15 mm^2^ or if bone marrow stimulation fails.^[2,15]^ Cartilage repair strategies are usually used for small lesions.^[16]^ Cartilage regeneration strategies are usually used for large lesions (>15 mm^2^).^[8]^ For patients with talar cartilage displacement and separation, the treatment of cartilage replacement strategies such as osteochondral autografts and allografts, juvenile articular cartilage allograft, and autologous bone marrow aspirate concentration.^[17]^

In cases where patients have severe OLT and the talar collapses due to necrosis, surgeons may perform talar surface replacement or total ankle replacement (TAA). While TAA can help maintain ankle function, it is generally more suitable for patients in advanced stages of arthritis.^[18]^

### 4.2. Option of arthroscopic debridement and microfracture, cartilage transplantation and partial talar surface prosthesis replacement in our study

Studies have shown that for large area OLTDs only to arthroscopic debridement and microfracture is usually satisfactory in the short term, most patients may eventually require secondary surgery, such as cartilage transplantation.^[19]^ Cartilage transplantation can cause further damage to the patient’s cartilage. From a clinical perspective, it has been observed that patients who undergo microfracture surgery with lesion areas >15 mm^2^, may not experience significant relief of symptoms. Moreover, there is a possibility that OLT may continue to deteriorate.^[20]^ If talar cartilage transplantation is performed, it is necessary to sacrifice the patient’s own cartilage. This increases the risk of cartilage damage in the area where the cartilage is taken, and further raises the possibility of arthritis and eventually they may not have satisfactory outcomes.^[21]^

Our team proposed a new surgical method for replacing injured cartilage with polyethylene materials on the surface of the talar cartilage. The callus at the medial ankle osteotomy had formed well and the patient gradually resumed weight-bearing activities.

### 4.3. The choice of prosthetic material: polyethylene or metal

In our study, the 3D printed prosthesis consisted of UHMWPE surface prosthesis and titanium alloy porous bone trabecular pedestal prosthesis (Fig. 3). A metal pedestal prosthesis with porous enhanced bone in growth and increased stability of the prosthesis.^[22]^ However one of the main challenges faced by polyethylene surface prostheses is their limited longevity, primarily due to wear of prostheses. The wear of polyethylene generates debris that triggers an inflammatory response, leading to osteolysis around the prosthesis and ultimately causing loosening and failure of the prosthesis.^[23,24]^Huiskes et al^[25]^ study shows that a higher modulus of elasticity in the implanted artificial joint prosthesis material resulted in a greater “stress masking” effect, leading to increased bone resorption around the implant and hindering early stabilization. A set of studies investigating the influence of different modulus of elasticity of implanted materials on bone resorption indicated that low modulus of elasticity materials can decrease bone resorption by 30% to 50% compared to high modulus of elasticity materials.^[25,26]^ Thus, the utilization of a material with a low modulus of elasticity could help reduce the “stress masking” effect. The mechanical properties of the contact interface, as well as the friction and wear characteristics of artificial joint materials, are factors critical to their performance. Polyethylene prostheses, in comparison to metal ones, exhibit attributes more similar to those of cartilage and exhibit enhanced cushioning effects during ankle movement.^[27]^

Therefore, we contend that the UHMWPE surface prosthesis is as applicable as a metal prosthesis. In our subsequent study, we plan to use a metal prosthesis if intraoperative circumstances are allowed and perform a comparative analysis.

### 4.4. Partial talar surface versus partial talus replacement and TAA

A case report indicated a customized 3D printed implant enabled treatment of the partial talus necrosis and collapse has favorable clinical outcomes after a 2-years follow-up.^[28]^ Another study reported that 2 patients with talar surface necrosis were treated after bionic 3D printing of talar surface prosthesis replacement, ankle pain was significantly reduced, ankle motion was satisfactory, and the distal articular surface of the tibia was not significantly accelerated.^[29]^ Ankle joint replacements for the large area of OLTs may lost excessive bone mass and suffered greater osteotomy and soft tissue injury.

Compared to existing partial talar prostheses and TAA, our design demonstrates: preserves enough bone mass that minimizes the need for bone amputation during the procedure and provides a range of options for later renovation surgery. Our teams prosthesis surface was made of UHMWPE material, whose elastic modulus more closely resembles cartilage and demonstrates superior wear resistance compared to all-metal prostheses. This procedure reduces the likelihood of ankle arthrodesis and preserves ankle joint function. It may be a preferable option particularly applicable especially for young and active individuals with a high demand for sport-related activities without extensive talar cartilage necrosis.

## 5. Conclusion

The 3D printed PTSR is a new surgical method for OLTD with a larger damage area that can provide satisfactory outcomes. Definitive and convincing conclusions are difficult to draw owing to the limited number of cases in this study. Another limitation is the follow-up period, which was 2 year. In addition, medium- and long-term follow-up is required. For this patient, we planned an annual follow-up, which included physical evaluation and radiography. In future, a prospective randomized controlled study should be conducted to manage the clinical surgical treatment of OLTDs including metal partial replacements.

## Author contributions

**Conceptualization:** Zhi Zou, Chuntao Xu, Zehui Jiang.

**Data curation:** Zhi Zou, Chuntao Xu, Zehui Jiang.

**Formal analysis:** Zhi Zou, Zehui Jiang.

**Funding acquisition:** Zhi Zou.

**Investigation:** Zhi Zou, Chuntao Xu.

**Methodology:** Zhi Zou.

**Project administration:** Lin Zou.

**Resources:** Zhi Zou, Chuntao Xu

**Software:** Zhi Zou.

**Supervision:** Zhi Zou, Chuntao Xu.

**Validation:** Zhi Zou.

**Visualization:** Zhi Zou.

**Writing – original draft:** Zhi Zou, Chuntao Xu.

**Writing – review & editing:** Zhi Zou, Lin Zou.
